# Cellular, mitochondrial and molecular alterations associate with early left ventricular diastolic dysfunction in a porcine model of diabetic metabolic derangement

**DOI:** 10.1038/s41598-020-68637-4

**Published:** 2020-08-06

**Authors:** Ilkka Heinonen, Oana Sorop, Bas M. van Dalen, Rob C. I. Wüst, Jens van de Wouw, Vincent J. de Beer, Yanti Octavia, Richard W. B. van Duin, Youri Hoogstrate, Lau Blonden, Milla Alkio, Katja Anttila, Andrew Stubbs, Jolanda van der Velden, Daphne Merkus, Dirk J. Duncker

**Affiliations:** 1 Division of Experimental Cardiology, Department of Cardiology, Thoraxcenter, Cardiovascular Research School COEUR, Erasmus University Medical Center, PO Box 2040, 3000 CA Rotterdam, The Netherlands; 2 Clinical Bioinformatics Unit, Department of Pathology, Erasmus University Medical Center, Rotterdam, The Netherlands; 3 Department of Urology, Erasmus University Medical Center, Rotterdam, The Netherlands; 4 Turku PET Centre, University of Turku, Turku, Finland; 5 Department of Clinical Physiology and Nuclear Medicine, University of Turku and Turku University Hospital, PO Box 52, 20521 Turku, Finland; 6 Department of Biology, University of Turku and Turku University Hospital, Turku, Finland; 7 Amsterdam UMC, Vrije Universiteit Amsterdam, Physiology, Amsterdam Cardiovascular Sciences, Amsterdam, The Netherlands; 8 Department of Human Movement Sciences, Faculty of Behavioural and Movement Sciences, Amsterdam Movement Sciences, VU University, Amsterdam, The Netherlands; 9 Netherlands Heart Institute, Utrecht, The Netherlands; 10 Rydberg Laboratory of Applied Sciences, University of Halmstad, Halmstad, Sweden; 11 Walter Brendel Center of Experimental Medicine (WBex), LMU Munich, 81377 Munich, Germany; 12 German Center for Cardiovascular Research (DZHK), Partner Site Munich, Munich Heart Alliance (MHA), 81377 Munich, Germany

**Keywords:** Cardiac hypertrophy, Heart failure

## Abstract

The prevalence of diabetic metabolic derangement (DMetD) has increased dramatically over the last decades. Although there is increasing evidence that DMetD is associated with cardiac dysfunction, the early DMetD-induced myocardial alterations remain incompletely understood. Here, we studied early DMetD-related cardiac changes in a clinically relevant large animal model. DMetD was established in adult male Göttingen miniswine by streptozotocin injections and a high-fat, high-sugar diet, while control animals remained on normal pig chow. Five months later left ventricular (LV) function was assessed by echocardiography and hemodynamic measurements, followed by comprehensive biochemical, molecular and histological analyses. Robust DMetD developed, evidenced by hyperglycemia, hypercholesterolemia and hypertriglyceridemia. DMetD resulted in altered LV nitroso-redox balance, increased superoxide production—principally due to endothelial nitric oxide synthase (eNOS) uncoupling—reduced nitric oxide (NO) production, alterations in myocardial gene-expression—particularly genes related to glucose and fatty acid metabolism—and mitochondrial dysfunction. These abnormalities were accompanied by increased passive force of isolated cardiomyocytes, and impaired LV diastolic function, evidenced by reduced LV peak untwist velocity and increased E/e′. However, LV weight, volume, collagen content, and cardiomyocyte cross-sectional area were unchanged at this stage of DMetD. In conclusion, DMetD, in a clinically relevant large-animal model results in myocardial oxidative stress, eNOS uncoupling and reduced NO production, together with an altered metabolic gene expression profile and mitochondrial dysfunction. These molecular alterations are associated with stiffening of the cardiomyocytes and early diastolic dysfunction before any structural cardiac remodeling occurs. Therapies should be directed to ameliorate these early DMetD-induced myocardial changes to prevent the development of overt cardiac failure.

According to a recent report by World Health Organization, age-standardized global prevalence of diabetes has nearly doubled since 1980, rising from 4.7% to 8.5% in the adult population.^1^ An estimated 422 million adults suffered from diabetes mellitus in 2014, compared to 108 million in 1980, which is the result of concomitantly increased prevalence of obesity and overweight globally^1^. As diabetes is associated with increased risk of cardiovascular diseases in particular, a deeper understanding of its cardiac pathophysiology is essential for the development of novel targeted treatments. Previous pre-clinical studies have addressed the consequences of diabetes in myocardial tissue, showing that the pathophysiology of diabetes-induced cardiac damage is a complex and multifactorial process, in which oxidative stress has been postulated as a key player^2–6^. Indeed, increased reactive oxygen species production and reduced antioxidant defenses have been associated with diabetic cardiomyopathy^2–5^. However, the majority of these studies have been performed in rodents^2–4^ and it remains uncertain whether these findings can be translated to a larger, human-like heart.

Consequently, in the present study we employed a translationally relevant large animal model with Diabetic Metabolic Derangement (DMetD), having similar anatomic, physiologic and metabolic characteristics of the cardiovascular system with the human situation. We characterized, in adult swine, the early DMetD-induced left ventricular (LV) changes at the mRNA, protein, cellular, tissue and organ function levels. For this purpose, DMetD was produced in adult male Göttingen miniswine by streptozotocin injections, to result in partial destruction of the pancreatic β-cells leading to impaired insulin production, combined with a high-fat, high sucrose and high fructose diet. LV function was assessed at baseline and 5 months after induction of DMetD, and myocardial tissue was comprehensively analyzed for oxidative stress and nitric oxide (NO) production, mitochondrial function, cardiomyocyte function, collagen content, and global LV remodeling and function, as well as for genome-wide gene expression profile. Our study shows that DMetD results in myocardial oxidative stress, endothelial nitric oxide synthase (eNOS) uncoupling and reduced NO production together with mitochondrial dysfunction and alterations in metabolic gene expression profile. These abnormalities are associated with impaired LV relaxation, at a time when cardiac structural remodeling at either the global LV or myocardial tissue level is still absent.

## Methods

### Animals and DMetD induction

Studies were performed in accordance with the NIH Guide for the Care and Use of Laboratory Animals (8th edition, National Research Council. Washington, DC: The National Academies Press, 2011) and were approved by the Animal Care Committee at Erasmus University Medical Center, Rotterdam, The Netherlands. A total of 17 adult male Göttingen minipigs were enrolled in the study and followed for 5 months. Diabetes was induced in 9 swine with intravenous injections of streptozotocin (25 mg/kg/day), over three days^7^. One week later, a high fat and high sugar diet (25% saturated fats, 1% cholesterol, 10% sucrose and 15% fructose) was gradually introduced to the diabetic swine (DMetD group, N = 9), whereas the healthy control swine (Control, N = 8) continued on normal pig chow. Swine were group-housed with a separate individual access to food for 1 h/meal, twice daily, and ad libitum access to water. Fasting mixed central venous blood samples were obtained at baseline and sacrifice (5-month time point), and analyzed for glucose, triglyceride and cholesterol.

### Fitness test

To objectively measure physical fitness, animals were subjected to an incremental endurance treadmill test until exhaustion. The test started at a pace of 1.5 km/h and speed was increased by 0.5 km/h every five minutes and running time was recorded as an indicator of maximal aerobic endurance capacity.

### Echocardiography

Echocardiography was performed at baseline, and after 5 months. Animals were sedated with an intramuscular injection of Zoletil (tiletamine/zolazepam; 5 mg/kg) and xylazine (2.25 mg/kg). Two-dimensional echocardiographic images (iE33, Philips, Best, the Netherlands) were acquired in harmonic mode from a right lateral decubitus position using a broadband (1–5 MHz) X5-1 transducer. All acquisitions and measurements were performed according to the current guidelines^8^. We performed pulsed-wave Doppler examination from the (apical) 4-chamber view, to obtain peak mitral inflow velocities at early (E) and late (A) diastole and E deceleration time. Tissue Doppler imaging was performed to measure myocardial tissue velocity at the septal and lateral mitral annulus at early diastole (e′). E/A ratio and the E/e′ ratio were calculated. Left atrial volume was measured using the modified biplane area-length method. Left atrial volume index was calculated by dividing left atrial volume by body weight.

### Hemodynamic assessments and cardiac tissue sampling and analyses

At 5 months follow up, hemodynamic measurements were perfomed under anesthesia and animals were terminated. Sedation was induced with Zoletil (tiletamine/zolazepam; 5 mg/kg), xylazine (2.25 mg/kg) and atropine (2 ml i.m.), and animals were anesthetized with an intravenous continuous infusion of pentobarbital (20 mg/kg), intubated and artificially ventilated. Arterial and venous access was obtained by placing 9F sheaths in the left carotid artery and the jugular vein for the measurement of mean aortic pressure, LV pressure (using a Millar catheter), as well as pulmonary artery and pulmonary capillary wedge pressure, cardiac output (by thermodilution) and for blood sampling. After placing a pressure-volume catheter in the LV (PV loop catheter, CD Leycom, The Netherlands), the pressure–volume loops were recorded and end-diastolic pressure–volume relationships were constructed, during baseline hemodynamic conditions, preload reduction (by complete obstruction of the inferior vena cava by a Fogarty balloon, 8/10F, Edwards Life sciences, Amsterdam, The Netherlands) and preload increase (by saline infusion, 20 ml/kg i.v. within 7 min). Thereafter, a sternotomy was performed, hearts were arrested, and quickly excised, washed in cold saline solution and then rapidly cut and frozen in liquid nitrogen and prepared and stored for later analyses.

### Myocardial reactive oxygen species (ROS) and nitric oxide (NO) production measurements

Cardiac oxidative stress was evaluated by lucigenin-enhanced chemiluminescence (Sigma Aldrich; 5 μmol/l) as previously described^7^. To this end, both basal and NADPH-stimulated (300 μM NADPH) superoxide generation were measured in homogenized, frozen sub-endocardial tissue samples of the anterior LV wall. NOS-dependent superoxide production was determined by incubating the samples for 20 min with the NOS inhibitor L-NAME (Sigma Aldrich; 1 mmol/l), while the contribution of NADPH oxidase to superoxide production was assessed using the NADPH oxidase inhibitor VAS2870, (10 μM). The temperature was controlled (37 °C) during the entire experiment and the measured light emission was expressed as relative light units (RLU) per mg protein per second. All samples were measured in duplicate and data averaged for each animal.

Aditionally, myocardial NO production was evaluated in the same area, by measuring the production of NO metabolites NO_2_^-^ and NO_3_^-^ using the Griess reaction colorimetric assay kit (Cayman Chemical).

### Endothelial nitric oxide synthase expression and phosphorylation

Protein expression of endothelial nitric oxide synthase (eNOS) and the phosphorylated eNOS were determined in homogenized, snap frozen LV sub-endocardial tissue samples. Furthermore, low temperature SDS-PAGE was performed for the detection of eNOS monomer and dimer fraction, as previously described^9^. SDS-PAGE for phosphorylated eNOS, total eNOS protein content and housekeeping protein glyceraldehyde 3-phosphate dehydrogenase (GAPDH) was performed at room temperature. Subsequently, proteins were transferred onto nitrocellulose membranes and the blots were probed with primary anti-phospho eNOS (1:1,000, Cell Signaling), anti-eNOS (1:500, Transduction Laboratory) and anti-GAPDH (1:10,000, Imgenex). All blots were analysed using the Odyssey system (LI-COR).

### AMPK western blot analyses

Frozen LV samples were weighed and homogenized in 6 vol ice cold 62.5 mM Tris–HCl buffer (pH 6.8, with 1 μg/ml Leupeptin, 1 μg/ml Pepstatin, and 1 mM phenylmethanesulfonyl fluoride, Sigma-Aldrich, St. Louis, Missouri, USA) with Qiagen TissueLyser (85220, Hilden, Germany). The homogenates were centrifuged and the samples were then denatured in Laemmli solution at + 70 °C for 7 mins^10^. Protein concentration of samples was determined using BCA protein assay kit (Thermo Scientific, Waltham, Massachusetts, USA). The samples (10 μg of protein for antibodies AMPK and pAMPK) were run with SDS-PAGE gels. After separation, the proteins were transferred onto a Whatman Protran Nitrocellulose membrane (PerkinElmer, Boston, Massachusetts, USA). The immunoblot membranes were blocked with 5% bovine serum albumin (BSA, Sigma-Aldrich) in Tris buffered saline (TBS, Sigma-Aldrich and thereafter, the membranes where incubated overnight at + 4 °C with primary antibodies for AMPK and pAMPK, washed and then incubated for 30 min with horseradish peroxidase conjugated secondary anti-rabbit antibody (1:10,000, Abcam, ab6721). The band densities were analysed with Gel Doc XR System (BioRad). In order to avoid variation between membranes a control sample was loaded onto each gel and the band intensities were normalized against the control sample.

### Enzyme assays

The samples were weighed and homogenized with 10 volumes of ice-cold homogenization solution (50 mM imidazole, 1 mM EDTA, pH 8.0) for HOAD (EC 1.1.1.35) or with 19 volumes homogenization buffer (50 mM Hepes, 1 mM, EDTA, 0.1% Triton X-100, pH 7.4) for CS (EC 2.3.3.1) and Lactate Dehydrogenase (LDH) (EC 1.1.1.27) with Qiagen TissueLyser. The HOAD samples were diluted 1:1 with homogenization solution, the LDH samples were diluted 1:1 with 50 mM Tris buffer, pH 7.4 and the CS samples were diluted 1:8 with 50 mM Tris buffer, pH 8.0. The protein concentrations of samples were determined with a BCA protein assay kit following the manufacturer´s protocol. The enzyme activity analyses were performed according to Dalziel et al.^11^ for CS and LDH and according to Lowry et al.^12^ for HOAD at 37 °C. The activities were read with PerkinElmer Enspire 2,300 Multilaber reader (Turku, Finland), and the enzyme activities were calculated per mg protein with the background activity subtracted.

### Histology and immunohistochemistry

After the heart was excised, LV myocardial tissue samples were fixated in 4% buffered formaldehyde, and embedded in paraffin. Thereafter, 4.5 μm thick slides were cut from the LV anterior wall, deparaffinized and stained for various histological analyses. For each analysis, 6–10 fields at × 20 magnification, were examined in the sub-endocardium of each slide. Collagen deposition was quantified using Picrosirius Red staining as follows: using a linear polarization filter, the area occupied by the collagen type I and type III fibers, was measured and expressed as percentage of the myocardial area. Myocyte size was quantified with a Gomori silver stain: cross-sectional areas of 300–350 round cells with clearly visible nuclei were measured in each slide. All measurements were performed using the Clemex Vision Image analysis system (Clemex Technologies, Quebec, Canada).

### Passive cardiomyocyte stiffness and titin isoform composition

Passive stiffness of single cardiomyocytes was measured using membrane-permeabilized cardiomyocytes. In brief, single cardiomyocytes were isolated in cold relaxing solution containing (in mM) free Mg^2+^ 1, KCl 100, EGTA 2, Mg-ATP 4, imidazole 10 (pH 7.0, adjusted with KOH) and incubated for 5 min in relaxing solution containing Triton X-100 (0.5%) to remove all membranes as described previously^13^. Passive stiffness of the cardiomyocytes, was assesed by *F*pas measurements, performed in relaxing solution at 15 °C and at sarcomere lengths ranging from 1.8 to 2.2 μm. Cardiomyocyte diameters were measured microscopically, in two perpendicular directions, and cross-sectional area was calculated assuming an elliptical shape. Passive force data were normalized to cross-sectional area of cardiomyocytes. Titin isoforms were separated on 1% agarose gel and stained with SYPRO Ruby protein stain as described previously^14,15^.

### Electron microscopy analysis of mitochondria

LV samples of 6 Control and 5 DMetD animals were prepared for electron microscopy as previously described^16^. In short, samples were fixed in 1.5% osmium tetroxide (10 min), dehydrated with acetone, and embedded in Epon812. Ultrathin sections were cut and placed on 300-mesh Formavar-coated nickel grids, and stained with uranyl acetate and lead citrate. Images from the intramyofibrillar region (longitudinal to the fiber orientation only) were taken at magnifications ranging from 4,500 × to 30,000 × with an electron microscope (Jeol-1200EX, Jeol Peabody, MA, USA). Glycogen content and the number of vacuoles were determined by scoring images on a scale for 1 (low) to 5 (extremely high) in a blinded fashion. Mitochondrial volume density was determined by overlaying a dense grid over images taken at 22,500 × and counting all mitochondria overlaying grid corners, as a percentage of all grid corners. Mitochondrial connectivity was assessed by visually inspecting whether mitochondria were touching other mitochondria, and expressed as a percentage of all mitochondria on the image. On average, 8 ± 1 images were analyzed per animal.

### Mitochondrial respiration

Mitochondrial respiration was measured in fresh biopsies from the LV sub-endocardium as described before^16^. Thin strips were permeabilised with saponin (50 µg/ml) for 30 min at 4 °C in a solution consisting of 7.2 mM EGTA, 2.8 mMCaEGTA, 6.6 mM MgCl_2_, 10 mM taurine, 5.8 mM ATP, 15 mM phosphocreatine, 20 mM imidazole, 50 mM MES and 0.5 mM DTT, and the pH was set to 7.1. The tissue was washed in respiration solution, consisting of 110 mM sucrose, 0.5 mM EGTA, 17 mM MgCl_2_, 20 mM taurine, 60 mM K-lactobionate, 10 mM KH_2_PO_4_, 20 mM HEPES, 1 g/L BSA (fatty acid free), and pH set to 7.1 before being transferred to a respirometer (Oxygraph-2 k; Oroboros Instruments, Innsbruck, Austria). Oxygen concentration remained < 300 μM during the experiment and temperature was set to 37 °C.

Leak respiration was measured during 10 mM sodium glutamate, 0.5 mM sodium malate and 5 mM sodium pyruvate. NADH-linked (through complex I) respiration was assessed using 2.5 mM ADP. Maximal NADH-linked respiration was measured after 10 μM cytochrome c, i.e. after correcting for possible outer-membrane damage. Maximal oxidative phosphorylation capacity was determined after 10 mM succinate. Maximal uncoupled respiration was assessed after titrations of 0.01 μM carbonylcyanide-4-(trifluoromethoxy)-phenylhydrazone (FCCP). Succinate-driven (complex II) respiration was assessed by inhibiting mitochondrial complex I by 0.5 μM rotenone. Residual oxygen consumption was measured after 2.5 μM antimycin A and used for background correction. Experiments were performed in duplo. Respiration values were averaged and normalized to wet weight and expressed in pmol O_2_/s/mg.

### Mitochondrial complex protein determinations by Western immunoblotting

Protein content was determined using western immunoblotting. Tissue homogenates containing 7.5 or 12.5 µg (well within the linear range of detection) of protein were loaded onto a pre-cast 4–15% or 8–16% gradient criterion TGX SDS-PAGE gels. Proteins were transferred onto PVDF membranes and incubated with an antibody cocktail (Abcam, Cambridge, UK; dilution 1:1,000; 2 h) against complex I subunit (NDUFB8), 30 kDa complex II subunit and complex IV subunit 1. Complex III subunit core 2 could not be determined due to species differences for the specificity of antibodies.

### RNA extraction and gene expression analyses

From 20 mg snap-frozen sub-endocardial tissue samples of 3 Control and 3 DMetD pigs, total RNA was extracted using the Qiagen miRNeasy Mini kit protocol. RNA integrity was checked on an Agilent 2,100 Bioanalyser for a RIN score ≥ 8.0 and 20 μg RNA at 100 ng/μl was used for further analyses. At the Biomics center of Erasmus University Medical Center, RNA was prepared for sequencing with the Illumina TrueSeq RNA sample preparation kit. Sequencing was performed according to the Illumina TrueSeq v3 protocol on an Illumina HiSeq 2000 sequencing system, 43 bp single read, 7 bp index. Sequence data were mapped against the reference pig genome *Sus scrofa* sequence assembly version 10.2 by Illumina Tophat version 2.0.10. Gene expression values of the RNA-seq data were estimated using featureCounts^17^ using the gene annotation Sscrofa10.2. Statistical differences in gene expression between both conditions were estimated using edgeR^18^ where an absolute logFC > 1 with a P-value < 0.001 was considered statistically significant. Biological functions and molecular networks of the differentially expressed genes were determined using Ingenuity pathway analysis Pathway analysis (Ingenuity Systems, Redwood City, CA, USA). Interconnectivity of the genes was visualized by the molecular networks constructed by the program.

### Data analysis

Data are presented as mean ± SEM. Comparison of variables between the DMetD and Control animals over time was performed by two-way ANOVA for repeated measures (fitness, echocardiography, PV-loop, myocyte force and blood variables) and Bonferroni post-hoc test or unpaired student t-test (variables measured only once at sacrifice) by GraphPad Prism 4.3 or SAS 9.2. *p* < 0.05 was considered statistically significant.

### Ethics approval and consent to participate

Studies were approved by the Animal Care Committee at Erasmus University Medical Center, Rotterdam, The Netherlands.

### Consent for publication

All authors have read and approved submission of the final version of the manuscript.

## Results

### Metabolic parameters and fitness

Several results are presented in the Supplementary Information file. Five months of DMetD resulted in hyperglycemia (p < 0.05), hypercholesterolemia (p < 0.05) and hypertriglyceridemia (p < 0.05), while no significant differences in body weight were observed between the groups (Table 1). DMetD resulted in a significant reduction in physical fitness, as reflected in a ~ 30% decrease in total running time (p < 0.05 versus Control).

**Table 1:** Body weight (BW) and plasma metabolic parameters measured at baseline and 5-month time points.

		Baseline	5-month
BW (kg)	CON	32 ± 2	37 ± 1
	DMetD	31 ± 2	40 ± 3^‡^
Glucose (mmol/l)	CON	5.9 ± 0.4	6.8 ± 0.7
	DMetD	5.0 ± 0.3	13.9 ± 2.0*^†‡^
Cholesterol (mmol/l)	CON	1.01 ± 0.13	1.10 ± 0.04
	DMetD	0.98 ± 0.05	5.89 ± 1.03*^†‡^
LDL cholesterol (mmol/l)	CON	0.45 ± 0.09	0.39 ± 0.03
	DMetD	0.42 ± 0.03	3.68 ± 1.05*^†‡^
Triglycerides (mmol/l)	CON	0.30 ± 0.02	0.27 ± 0.03
	DMetD	0.26 ± 0.03	0.66 ± 0.19*^†‡^
Fitness test (min)	CON	39 ± 4	38 ± 4
	DMetD	43 ± 5	30 ± 4*^‡^

*CON* healthy controls (n = 8), *DMetD* diabetic metabolic derangement animals (n = 9), *LDL* low density lipoproteins

*p < 0.05 as time·diabetes interaction by two-way ANOVA

^†^p < 0.05 versus corresponding CON by Bonferroni post-hoc analysis

^‡^p < 0.05 versus corresponding baseline by Bonferroni post-hoc analysis. Data are mean ± SEM.

### Cardiac function and remodeling

**Figure 1:**
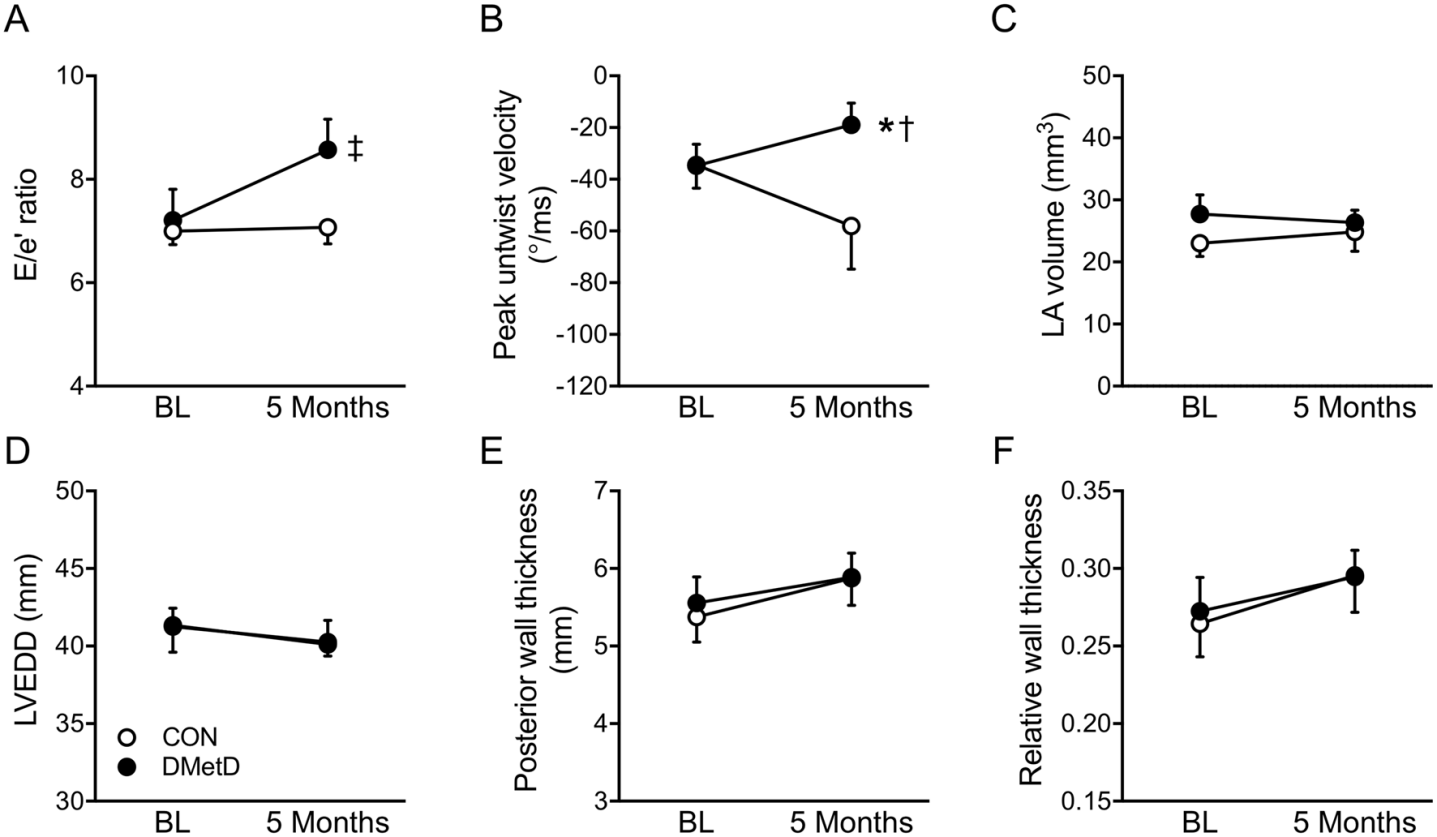
Ratio of mitral peak velocity during early filling (E) to early diastolic mitral annular velocity (e′, E/e′ ratio, **A**), peak left ventricle untwist velocity (**B**), left atrial volume (LA, **C**), left ventricle end diastolic diameter (LVEDD, **D**), posterior wall thickness (PWd, **E**), and relative wall thickness ((2*PWd)/LVEDD, **F**) in the hearts of DMetD and Control (CON) swine at baseline (BL) and after 5 months. *p < 0.05 for interaction DMetD and time by two-way ANOVA, ^†^p < 0.05 versus corresponding CON by Bonferroni post-hoc test, and ^‡^p < 0.05 versus CON by unpaired t-test.

**Figure 2:**
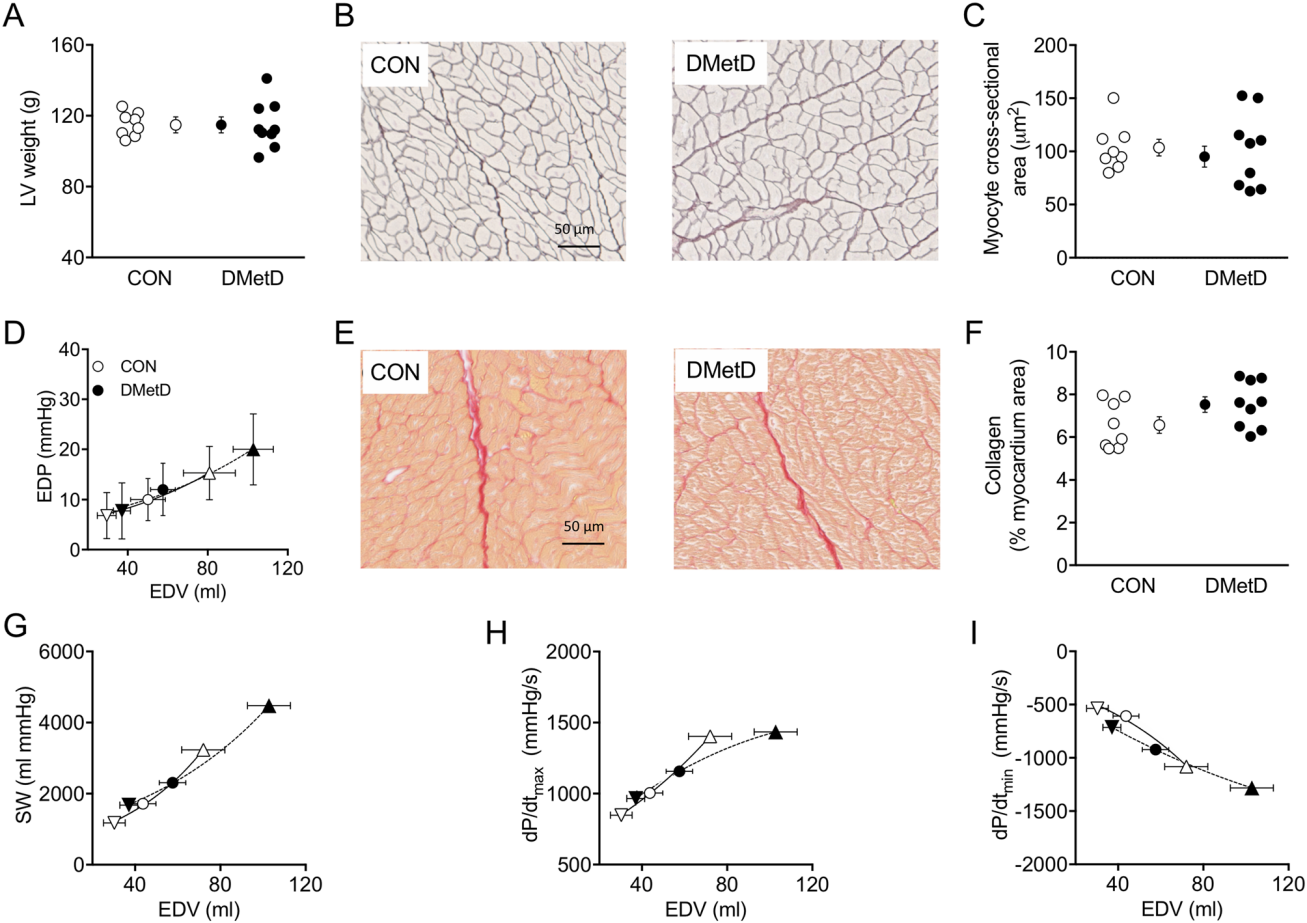
Left ventricular weight (LV weight, (**A**)), myocyte cross-sectional area (examples, (**B**), and data summary (**C**)), end-diastolic pressure (EDP)–volume (EDV) relation composed from measurements at preload reduction, (inverted triangle), baseline (open circle) and preload increase (open triangle) (**D**), myocardial collagen deposition (examples, (**E**) and data summary (**F**)), stroke work (SW, **G**), and dP/dt_max_ (**H**) and dP/dt_min_ (**I**), plotted as a function of end-diastolic volume, measured at sacrifice in CON and DMetD animals.

There were no differences in echocardiography variables between Control and DMetD at baseline (Tables S1 and S2). Five months of DMetD resulted in an elevated E/e’, while peak untwist velocity was significantly lower, in DMetD compared to Control (Fig. 1), indicating impaired LV diastolic function in DMetD. In contrast, DMetD did not produce differences in left atrial (LA) volume, LV diameter, or absolute and relative LV wall thickness between DMetD and Control (Fig. 1), while LV weight was also not affected (Fig. 2A), indicating that 5 months of DMetD did not result in cardiac remodeling. Moreover, there were no significant differences in LV and aortic pressures, LV volumes, stroke volume, ejection fraction, or cardiac output between DMetD and Control (Tables S3 and S4). Histological analysis showed that cardiomyocyte size (Fig. 2B, C) and myocardial collagen content (Fig. 2E, F) were not significantly different between DMetD and Control. Furthermore, there were no changes in type I or type III collagen or their ratio (data not shown). These histological findings correlated well with the nearly identical LV weights and LV end-diastolic pressure–volume relations, (Fig. 2D), the preload recruitable stroke work (Fig. 2G), and preload adjusted dP/dt_max_ (Fig. 2H) and dP/dt_min_ (Fig. 2I) in DMetD and Control.

### Isolated cardiomyocyte function

**Figure 3:**
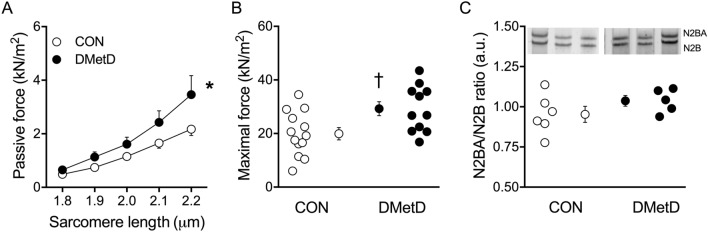
Single cardiomyocyte passive force (**A**) in response to increasing myocyte stretch and maximal forces (**B**). CON = controls (13 cardiomyocytes of 5 swine), DMetD, diabetic metabolic derangement (11 cardiomyocytes of 5 swine). Myocardial titin N2BA/N2B ratio of CON (n = 6) and DMetD (n = 5, C), and typical examples of the titin gels for 3 animals in each group . *p < 0.05 versus CON by two-way ANOVA, ^†^p < 0.05 versus CON by unpaired t-test. Full-length gels are shown in Supplementary Fig. S1.

Single cardiomyocyte passive force was significantly higher in the DMetD group compared to Control (Fig. 3A) as was the maximally developed force (Fig. 3B). No differences were observed in titin isoform composition evidenced by an unaltered ratio between the compliant (N2BA) and stiff (N2B) isoform of titin between DMetD and Control (Fig. 3C).

### Reactive oxygen species and nitric oxide

**Figure 4:**
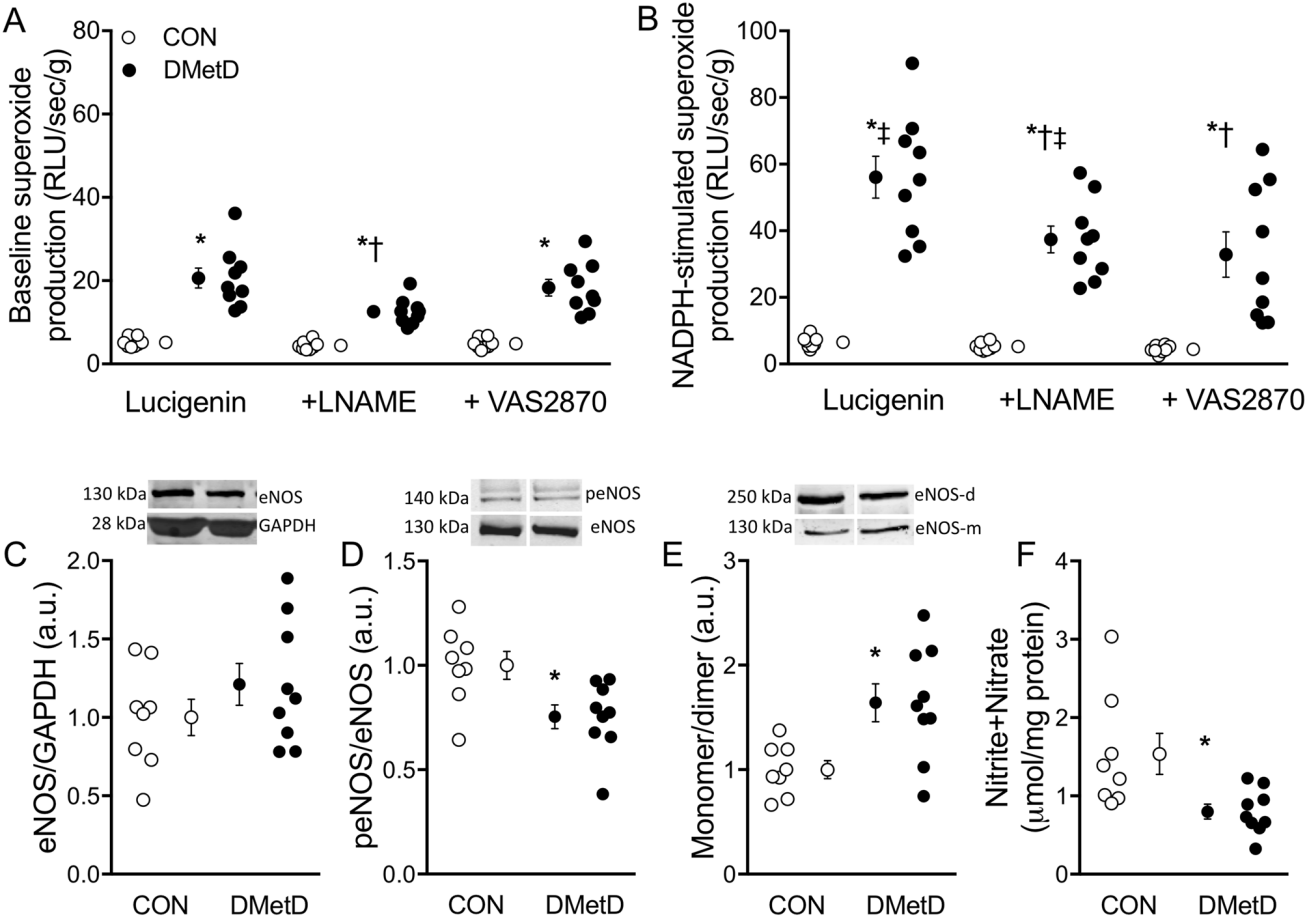
Basal (**A**) and NAPDH-stimulated (**B**) superoxide production in LV myocardium, myocardial eNOS expression (**C**), phosphorylation of eNOS (**D**), the monomer/dimer ratio (**E**), and nitric oxide (NO) production (**F**). CON = controls (n = 8), DMetD, diabetic metabolic derangement (n = 9). *p < 0.05 versus corresponding CON by two-way ANOVA and Bonferroni post hoc (panel **A** and **B**) or unpaired t-test (panel **C**–**F**), ^†^p < 0.05 versus corresponding untreated by two-way ANOVA and Bonferroni post hoc, ^‡^p < 0.05 versus corresponding basal by two-way ANOVA and Bonferroni post hoc. Full-length gel blots are shown in Supplementary Fig. S2.

Superoxide production in the LV myocardium was markedly higher in DMetD compared to Control under baseline conditions as well as during inhibition of NOS and NADPH oxidase (Fig. 4A). Furthermore, upon stimulation of NADPH oxidase (Fig. 4B), superoxide production was further enhanced in the DMetD group, suggesting that NADPH oxidase is a significant source of superoxide. This increase was attenuated by L-NAME, both under basal conditions (Fig. 4A), as well as under NADPH stimulation (Fig. 4B), suggesting that a significant part of the superoxide production is NOS-dependent. eNOS expression in DMetD was comparable to Control (Fig. 4C), but the ratio between phosphorylated and unphosphorylated eNOS was reduced in DMetD (Fig. 4D). Moreover, monomer-to-dimer ratio was also significantly increased, suggestive of eNOS uncoupling (Fig. 4E), and NO production was reduced in DMetD (Fig. 4F). The decrease in eNOS phosphorylation and increase in eNOS uncoupling correlated with the superoxide production (both p < 0.05), further supporting the contribution of eNOS uncoupling to the oxidative stress in the hearts of DMetD animals.

### Mitochondrial structure and function

**Figure 5:**
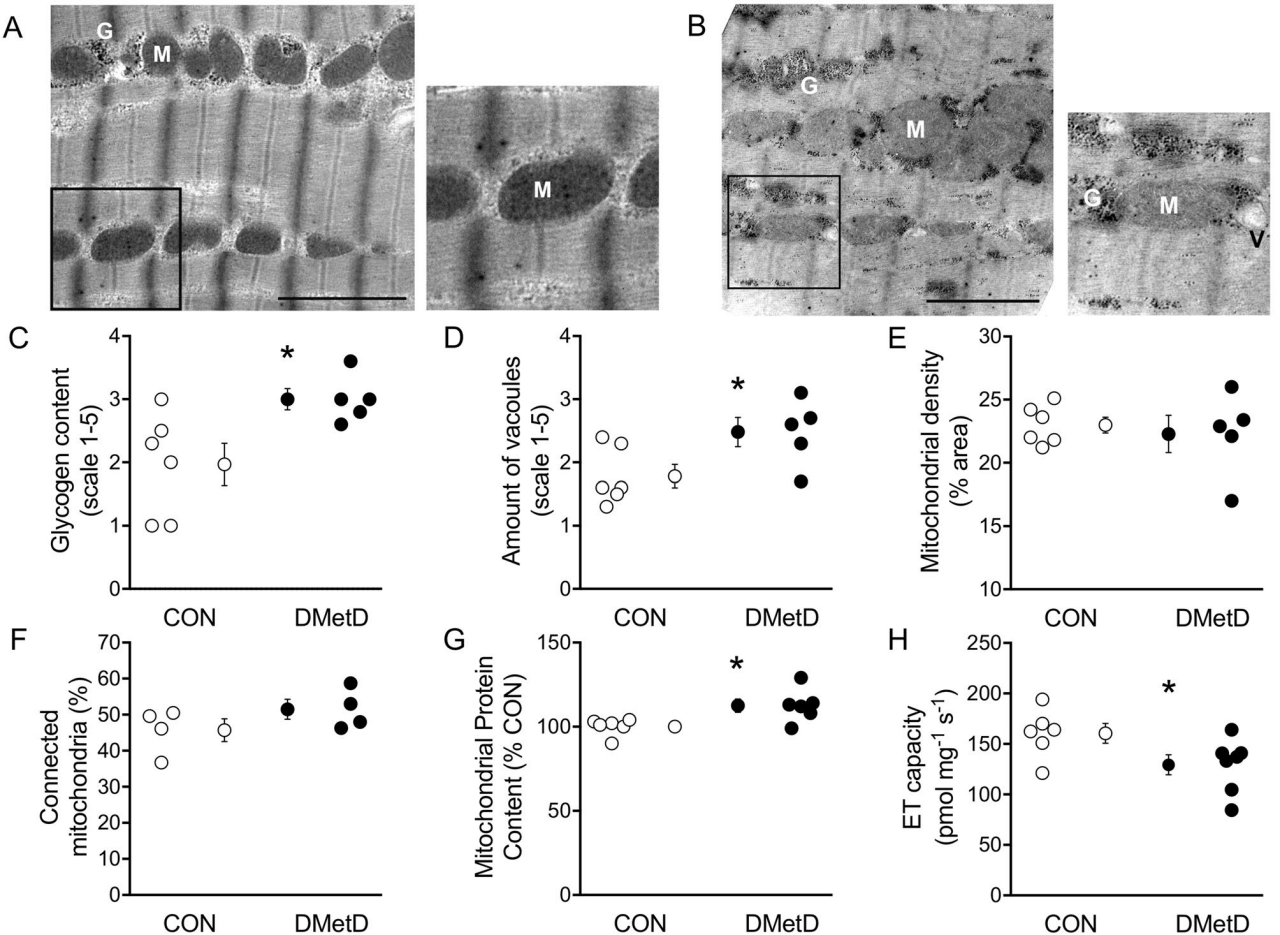
Examples of cardiomyocyte ultrastructure by electron microscopy in CON (**A**) and DMetD (**B**). M = mitochondrion, G = glycogen, V = vacuole. Scale bar represents 2 μm. The inserts highlight the altered glycogen content. Quantified glycogen content (**C**) and number of vacuoles (**D**) were significantly higher in DMetD compared to CON. Mitochondrial density (**E**) or percentage of connected mitochondria (**F**) were not different between groups. Total myocardial mitochondrial protein content (**G**) by Western blotting (see Supplementary Fig. 1) was significantly higher in DMetD compared to CON. Electron transport (ET) capacity (**H**) however, was significantly lower in DMetD compared to CON, due to NADH-linked dysfunction (see Supplementary Fig. 2), suggestive of an intrinsic mitochondrial dysfunction, independent of mitochondrial protein mass. *p < 0.05 *vs.* CON by unpaired t-test. Full-length gels and blots are shown in Supplementary Fig. S3.

Electron microscopy (EM) analysis of the cardiomyocytes (Fig. 5A, B) demonstrated increased glycogen content in the cardiomyocytes of DMetD compared to Control (Fig. 5C), indicating a clear diabetic phenotype. Additionally, more (and larger) vacuoles were observed in DMetD myocardium compared to Control (Fig. 5D), indicative of either fatty acid accumulation or enlarged sarcoplasmic reticulum. Mitochondrial density (Fig. 5E) and percentage of interconnected mitochondria (Fig. 5F) were similar in both groups, suggesting that the network connectivity remained intact in DMetD. Total myocardial mitochondrial complex protein content was slightly higher in DMetD (Fig. 5G and S3), but maximally uncoupled respiration (normalized to wet weight) was lower in DMetD compared to Control (− 20%, p < 0.05; Fig. 5H). This lower mitochondrial respiration could, at least in part, be explained by a lower maximal NADH (complex I)-stimulated respiration in DMetD compared to Control (− 31%, p = 0.02; Supplementary Fig. S4), while maximal complex II-linked respiration was similar (Fig S4), indicative of a mitochondrial complex I dysfunction in DMetD.

### AMPK and metabolic enzymes

**Figure 6:**
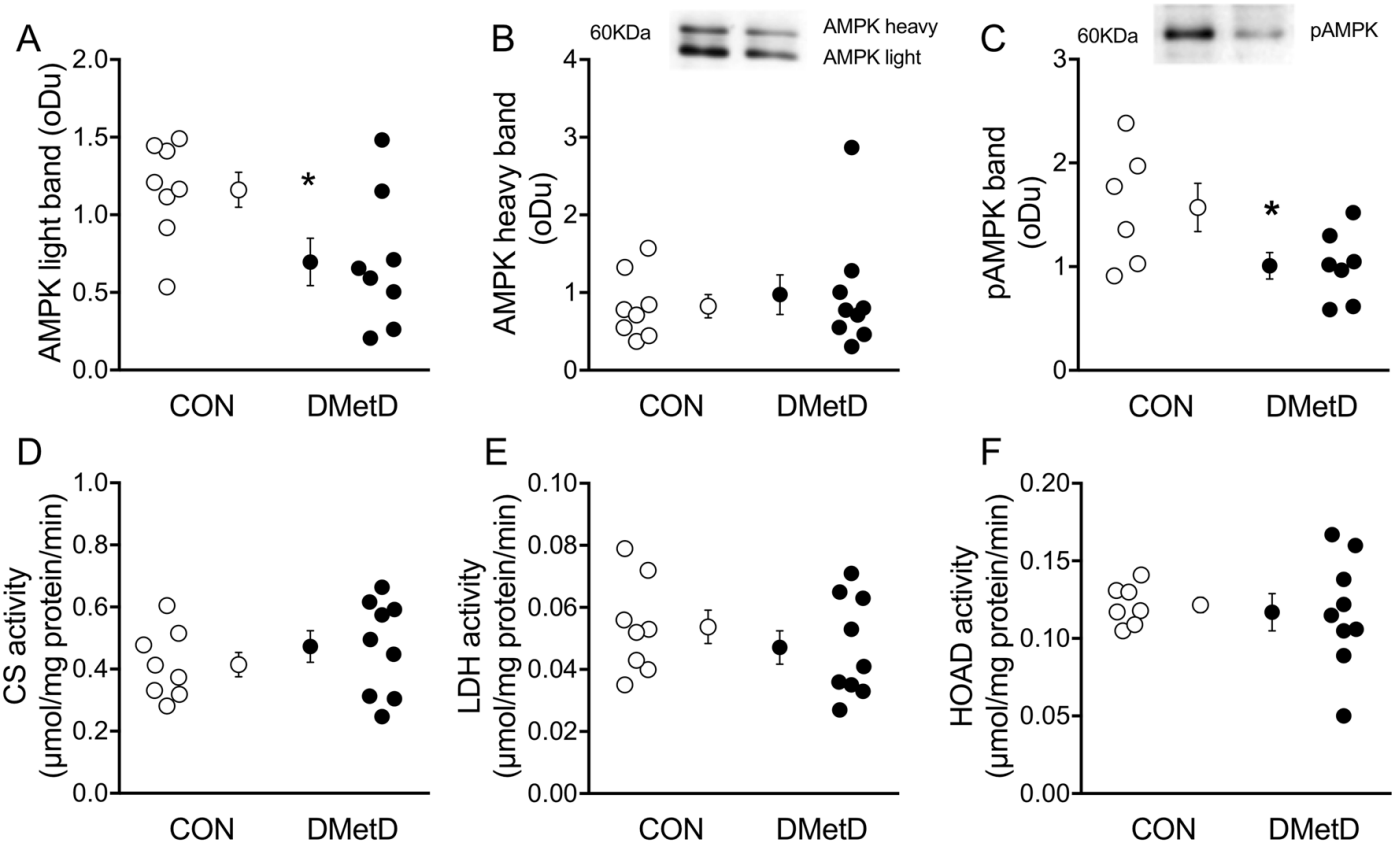
Protein levels of the AMPK light (**A**) and heavy bands (**B**), pAMPK (**C**) and enzyme activity of CS (**D**), LDH (**E**) and HOAD (**F**), in CON and DMetD animals. *p < 0.05 versus CON by unpaired t-test. *AMPK * AMP-activated protein kinase, *pAMPK* phosphorylated AMPK, *CS* citrate synthase, *LDH* lactate dehydrogenase, *HOAD* 3-hydroxyacyl-CoA dehydrogenase. Full-length gel blots are shown in Supplementary Fig. S5.

The analysis of AMPK light and heavy bands showed lower protein levels of the AMPK light bands in the DMetD animals (Fig. 6A), while no differences were observed in the heavy bands (Fig. 6B). The levels of pAMPK were also lower in the DMetD group (Fig. 6C). Enzyme activities of citrate synthase (CS), lactate dehydrogenase (LDH) and 3-OH acyl CoA dehydrogenase (HOAD) were similar between groups (Fig. 6D–F).

### Genome-wide gene expression

**Figure 7:**
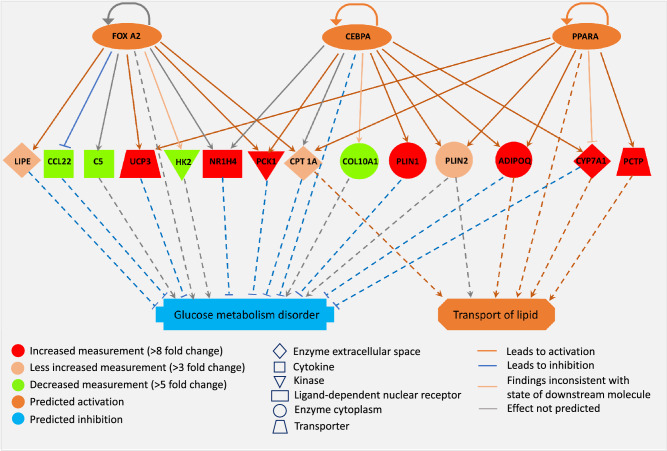
Myocardial genes and their networks in DMetD are connected to two major pathways associated with glucose and lipid metabolism disorders.

Genome-wide gene expression analyses indicated that transcription of 63 different genes were statistically significantly differentially expressed, with 29 genes up-regulated and 34 genes down-regulated by DMetD, of which many were related to alterations in glucose and fatty acid metabolism (Supplementary Table S6). The most significantly affected networks were ‘glucose metabolism disorder’ and ‘transport of lipids’ and within these networks we identified three transcriptional factors being involved, i.e. FOX A2, CEBPA and PPARA (Fig. 7).

## Discussion

The present study was designed to investigate the early cardiac functional and structural abnormalities produced by DMetD in a human-like, translational large animal model, at the level of gene and protein expression, cellular and tissue composition as well as organ function. The main findings were that: (*i*) After five months of DMetD, no changes in LV weight, LV end-diastolic-pressure, -volume, and -pressure–volume relation, and LA volume were observed. Similarly, no LV myocardial fibrosis or cardiomyocyte hypertrophy was observed, indicating the absence of overt remodeling at the LV chamber or myocardial tissue level. (*ii*) In contrast, cardiomyocyte resting tension was increased, which was accompanied by an elevated E/e’ and a lower LV peak untwist velocity in DMetD, reflecting early impairments in LV diastolic function. (*iii*) Mitochondrial dysfunction in DMetD swine was also present with lower mitochondrial complex I function and lower maximal respiration, despite minor changes in mitochondrial protein content or mitochondrial density. (*iv*) DMetD also resulted in marked perturbations in LV myocardial nitroso-redox balance, due to increased superoxide and reduced nitric oxide production. (*v*) These abnormalities were accompanied by differential expression of a significant number of genes, many of which related to perturbations in glucose and fatty acid metabolism. The implications of these findings will be discussed.

### DMetD-associated LV diastolic dysfunction in the absence of LV remodeling

DMetD is a major risk factor for heart failure, in particular heart failure with preserved ejection fraction (HFpEF)^19,20^. HFpEF is characterized by an increased LV diastolic stiffness and LV concentric remodeling, while at the myocardial ultrastructural level fibrosis and concentric myocyte hypertrophy are typically observed^21,22^. In the present study, 5 months of DMetD did result in robust hyperglycemia and dyslipidemia, with hypercholesterolemia and increased plasma triglycerides, in the absence of liver or kidney dysfunction (see Supplementary Table S5), consistent with our and others’ findings in similar animal models^7,23,24^. However, despite these clear metabolic alterations DMetD did not—yet—result in LV or cardiomyocyte hypertrophy or increased LV end-diastolic stiffness or produce myocardial fibrosis, suggesting that overt LV structural changes were still absent. Additionally, no macroscopic atherosclerosis development could be observed at this point in any of the large epicardial coronary arteries. In contrast, we observed several functional changes both in LV chamber and cardiomyocyte function. Thus, peak untwist velocity—a novel and early functional marker of LV diastolic dysfunction—was lower, while E/e′—an established marker of diastolic dysfunction—was elevated in DMetD versus Control swine at 5 months of DMetD. Interestingly, we recently found that a reduction of peak untwist velocity was already observed after 3 months of DMetD, at a time when E/e′ was still maintained normal^25^. The observation in the present study, that 5 months of DMetD produced a further reduction in peak untwist velocity and resulted in an elevated E/e′, suggests that diastolic dysfunction progresses over time. The described increase in E/e′ ratio and peak untwist velocity may be a reflection of the dysfunctional (subendocardial) cardiomyocytes as an early manifestation of diastolic dysfunction, before an overt increase in collagen deposition increases chamber stiffness more profoundly^26^. This may explain why LVEDP and LV end-diastolic elastance, as assessed with LV pressure–volume measurements, were still maintained at this stage of the disease. However, the lack of difference between DMetD and Control animals with respect to other early diastolic function variables, including Tau and LV dP/dtmin is not readily explained. One potential explanation is that echocardiography was performed under light sedation, while LV pressure–volume measurements were obtained under deep general anaesthesia with pentobarbital; the latter may have obscured subtle differences in early diastolic function parameters between DMetD and Control animals.

Indeed, the changes in LV diastolic function were accompanied by an increased passive force of single cardiomyocytes, suggesting that altered cardiomyocyte function, rather than structural changes, were responsible for the observed LV diastolic dysfunction. A higher resting cardiomyocyte tension has been shown in patients with HFpEF^21,27^ as well as in DMetD patients^28^ and animal models^7,29,30^. The increased resting tension has initially been ascribed to changes in titin isoform expression^31^. However, in line with more recent studies suggesting that changes in titin phosphorylation (rather than the isoform changes) are principally responsible for the increased resting tension^32,33^, we also failed to observe differences in titin isoform expression. There is increasing evidence that hypo-phosphorylation of titin is the result of reduced NO-cGMP-PKG signaling^27,33^. Interestingly, we observed a reduced nitric oxide production in DMetD swine, warranting further investigation of changes in titin phosphorylation in DMetD animals in future studies.

### Oxidative stress and impaired nitric oxide formation

Five months of DMetD resulted in substantially higher superoxide production in LV myocardium compared to Control swine. Superoxide production was already significantly higher at basal state, which was principally NOS mediated. In addition, superoxide production was further aggravated in DMetD myocardium upon exposure to NADPH, which appeared to be mediated by both NOS and NADPH oxidase. These findings are in line with other studies demonstrating oxidative stress in DMetD and suggest that a variety of mechanisms can contribute to oxidative stress in DMetD^34,35^. Moreover, the increased superoxide production was likely directly related to abnormalities in NO bio-availability. An increased NOS dependent superoxide production is consistent with our finding that monomer/dimer ratio of eNOS was significantly higher in DMetD myocardium. This suggests substantial eNOS uncoupling, which resulted in superoxide— rather than NO-production by eNOS. Moreover, although total eNOS was not different between the groups, the ratio of phosphorylated and unphosphorylated eNOS was reduced in DMetD, which may have been the result of the observed lower levels of AMPK and pAMPK^36,37^. In line with these findings, reduced NO levels, as indicated by the NO metabolites nitrite-and nitrate, were present in the DMetD myocardium. Reduced NO levels are not only detrimental for coronary vascular function^38^, but also for myocardial function^36,39^, and the loss of NO, and consequently NO-cGMP-PKG signaling, likely explains the observed increase in cardiomyocyte resting tension^27^.

### AMPK and enzymatic activities in the LV

We observed an inhibition or deactivation of the AMPK system in DMetD, as we measured lower AMPK phosphorylation and AMPK light band levels. AMPK is an important regulator of cellular energy pathways and is activated by stressful situations such as prolonged exercise when AMP/ADP ratio is elevated^37,40^. Unlike in rodents, in fish and swine two AMPK bands can be detected. The physiological meaning of these two bands has not been fully elucidated, but they may represent different isoforms of AMPK. The downregulation of AMPK was likely the result of the increased circulating glucose and lipid levels in conjunction with the higher tissue glycogen levels in DMetD swine, which was previously observed also in mouse models of DMetD^41,42^. A decrease in AMPK activity explains, at least in part, the increased NADPH oxidase activity as well as the reduced peNOS levels in DMetD swine, and thus likely contributed to the perturbations in nitroso-redox signaling and the consequent increase in cardiomyocyte resting tension^37^.

### Metabolic and mitochondrial function

Myocardial glycogen content was higher in DMetD, which is consistent with findings in previous studies^43,44^. The higher number of vacuoles in the DMetD LV myocardium likely reflects increased fat deposits, but could also be part of enlarged peroxisomes (for fatty acid oxidation) or sarcoplasmic reticulum. Unfortunately, delineation between these possibilities cannot be derived from our electron microscopy images. However, the lack of functional alterations in systolic cardiac function in DMetD suggests that the sarcoplasmic reticulum remains intact in this animal model and these enlarged vacuoles are likely related to lipid overload, and a state of lipotoxicity^6^.

Additionally, mitochondrial density and connectivity were not affected in DMetD, while total mitochondrial complex protein content was slightly higher. No significant alterations were detected in the maximal activity of myocardial enzymes essentially involved in cardiomyocyte energy generation, including CS, LDH and HOAD. Maximally stimulated mitochondrial respiration was lower in DMetD, due to a lower NADH-linked (complex I) respiration. Mitochondrial complex I is the most vulnerable complex for mitochondrial (supercomplex) damage, and dysfunction has been seen in other models of heart failure^16^, and type 2 diabetes mellitus^45^. Likely, the mitochondrial complex I dysfunction causes bioenergetic dysfunction and ADP insensitivity of the heart, contributing to ADP-induced stiffening of the heart^46^. The cause of this impaired mitochondrial complex I function is currently unknown, but could relate to local inflammation and/or lipotoxicity,^6,45^ or oxidative stress-induced alterations in supercomplex formation^6, 16^ that in turn can cause an increase in mitochondrial superoxide formation^47^. Further studies are required to understand the contribution of mitochondrial dysfunction and the role of oxidative stress in the development of diastolic dysfunction in DMetD.

### Gene expression profiles

Our genome-wide gene expression data analysis indicated that after 5 months of DMetD transcription of 364 different genes was either up-or down-regulated by DMetD by at least twofold. From these 364 genes two highly significant networks emerged that were related to abnormalities in glucose metabolism (glucose metabolism disorder) and fatty acid metabolism (transport of lipid). After p-value correction (FDR < 0.1), 63 genes remained statistically significant, of which several genes are particularly relevant in relation to the diabetic cardiomyopathy.

First, the upregulation of UCP3 (uncoupling protein 3) which results in dissipation of energy as heat, can protect mitochondria against lipotoxicity and lipid-induced oxidative stress, observed here. Expression levels of UCP3 increase when fatty acid supply to mitochondria exceeds their beta-oxidation capacity and the protein enables the export of excess fatty acid load from mitochondria^48^. Another upregulated gene, ANGPTL4, encodes for the protein Angiopoietin-like 4, of which expression is induced by low oxygen levels and is also directly involved in regulating lipid metabolism. In diabetes, increased expression leads to reduced triglyceride clearance in blood, and explains, at least in part, the observed hypertriglyceridemia in DMetD swine. Additionally, we observed upregulated CPT1A, carnitine palmitoyltransferase 1A, which has a rate-limiting role for long chain fatty acid oxidation in cardiac mitochondria. Its expression has been shown to be increased in hypertrophied rat cardiomyocytes^49^, resulting in impaired fatty acid oxidation^50^. Although the mechanisms responsible for such increase in the present study are unclear, and may be related to an impaired inhibition of malonyl-CoA by glucagon in the DMetD hearts, it may contribute to accumulation of fatty acid metabolites in the myocardium, contributing to the increased lipotoxicity^50^. Lipase E was also up-regulated in DMetD hearts, and is known to regulate hydrolysis of stored triglycerides to free fatty acids. Perilipin 2 (PLIN2) coats intracellular lipid storage droplets, and its up-regulation together with lipase E indicates enhanced free fatty acid traffic in the DMetD myocardium, and suggests that the higher number of vacuoles in DMetD indeed represent lipid accumulation. The protein encoded by the CREB3L3 is a transcription factor that may act during endoplasmic reticulum stress by activating unfolded protein response target genes, suggesting that endoplasmic reticulum stress is present in the DMetD myocardium^51^.

Out of the most down-regulated genes (largest fold-change), the physiological meaning of the protein encoded by GNMT gene (an enzyme that catalyzes the conversion of S-adenosyl-l-methionine to S-adenosyl-l-homocysteine and sarcosine) is currently unclear in the diabetic heart. The observed down-regulation of SLC2A1 gene, which normally provides instructions for producing glucose transporter protein type 1 (GLUT1) is consistent with reduced glucose uptake in diabetic myocardium^52^. Furthermore, HAPLN3 (Hyaluronan and proteoglycan link protein 3) has previously been associated with diabetes in epidemiological studies. The ALDH4A1 gene codes for the enzyme delta-1-pyrroline-5-carboxylate dehydrogenase, which is a mitochondrial matrix NAD-dependent dehydrogenase producing glutamate; its down-regulation confirms early impairments in NADH-linked respiration. Down-regulation of CYP4F55 (cytochrome P450) also suggests overall alterations in mitochondrial substrate oxidation, but the role of the cytochrome P450 system in the heart is largely unknown^53^.

Although we did not investigate the causal relation between the observed alterations in gene-expression and the cardiac phenotype in DMetD animals, it could be speculated that some of these genes, expecially those related to mitochondrial function, could serve as potential drug targets to treat diabetic cardiomyopathy. Future studies are therefore needed to determine the therapeutic potential of interfering with (or enhancing) the DMetD-induced alterations in gene-expression.

## Conclusions

The present study shows that diabetic metabolic derangement in a large animal model, with high resemblance to the human heart, resulted in myocardial oxidative stress, eNOS uncoupling and reduced NO production, together with an altered metabolic gene expression profile and mitochondrial dysfunction. These myocardial tissue alterations were associated with cardiomyocyte stiffening and early left ventricular diastolic dysfunction, before any overt structural cardiac remodeling occurs. Therapies should be directed to ameliorate these early DMetD-induced myocardial changes to prevent the development of overt cardiac failure.

## Supplementary information

Supplementary Information.

## Data Availability

The datasets generated during and/or analyzed during the current study are available from the corresponding author upon reasonable request.
