# Supplementary material for: Cellular, mitochondrial and molecular alterations associate with early left ventricular diastolic dysfunction in a porcine model of diabetic metabolic derangement
Source: Sci Rep. 2020 Aug 6;10:13173. doi: 10.1038/s41598-020-68637-4 (PMC7413251; doi:10.1038/s41598-020-68637-4)

Supplemental results- SREP-19-43514B

Cellular, mitochondrial and molecular alterations associate with early left ventricular diastolic dysfunction in a porcine model of diabetic metabolic derangement

Ilkka Heinonen[1,4,5,10,*], Oana Sorop[1], Bas Van Dalen[1], Rob C.I. Wüst[7,8], Jens van de Wouw[1], Vincent J. de Beer[1], Yanti Octavia[1], Richard W. B. van Duin[1], Youri Hoogstrate[3], Lau Blonden[1], Milla Alkio[6], Katja Anttila[6], Andrew Stubbs[2], Jolanda Van der Velden[7,9], Daphne Merkus[1] and Dirk J. Duncker[1,*]

[1]Division of Experimental Cardiology, Department of Cardiology, Thoraxcenter, Cardiovascular Research School COEUR, Erasmus University Medical Center, Rotterdam, The Netherlands. [2]Clinical Bioinformatics Unit, Department of Pathology, Erasmus University Medical Center, Rotterdam, The Netherlands. [3]Department of Urology, Erasmus University Medical Center, Rotterdam, The Netherlands. [4]Turku PET Centre, University of Turku, Turku Finland. [5]Department of Clinical Physiology and Nuclear Medicine, and [6]Department of Biology, University of Turku and Turku University Hospital, Turku, Finland. [7]Amsterdam UMC, Vrije Universiteit Amsterdam, Physiology, Amsterdam Cardiovascular Sciences, Amsterdam, The Netherlands. [8]Department of Human Movement Sciences, Faculty of Behavioural and Movement Sciences, Amsterdam Movement Sciences, VU University, Amsterdam, The Netherlands. [9]Netherlands Heart Institute, Utrecht, The Netherlands; [10]Rydberg Laboratory of Applied Sciences, University of Halmstad, Halmstad, Sweden.

***Corresponding authors**:

Ilkka Heinonen, Erasmus University Medical Center Rotterdam, PO Box 2040, 3000 CA Rotterdam, The Netherlands. Email: i.heinonen@erasmusmc.nl (current Address: University of Turku, PO Box 52, FI-20521 Turku, Finland. Email: ilkka.heinonen@utu.fi)

Dirk J. Duncker, Erasmus University Medical Center Rotterdam, PO Box 2040, 3000 CA, Rotterdam The

Netherlands, Email: d.duncker@erasmusmc.nl

Table S1 Body weight, left atrium and conventional ventricular echocardiography characteristics from baseline to 5-month follow-up.

		Baseline	5-MONTH
Body weight (kg)	CON	32 ± 2	37 ± 1
	DMetD	31 ± 2	40 ± 3
LA volume (mm^3)	CON	28 ± 3	26 ± 2
	DMetD	23 ± 2	25 ± 3
Relative LA volume (mm^3/kg)	CON	0.86 ± 0.07	0.71 ± 0.05
	DMetD	0.75 ± 0.06	0.63 ± 0.06
LVEDD (mm)	CON	41 ± 2	40 ± 1
	DMetD	41 ± 1	40 ± 1
Relative LVEDD (mm/kg)	CON	1.31 ± 0.26	1.10 ± 0.16
	DMetD	1.36 ± 0.02	1.05 ± 0.24
PWd (mm)	CON	5.4 ± 0.3	5.9 ± 0.4
	DMetD	5.6 ± 0.3	5.9 ± 0.3
IVSd (mm)	CON	5.9 ± 0.2	6.3 ± 0.4
	DMetD	5.9 ± 0.4	5.8 ± 0.3
E (cm/s)	CON	55 ± 3	51 ± 3
	DMetD	53 ± 3	53 ± 5
RWT (unitless)	CON	0.26 ± 0.02	0.30 ± 0.02
	DMetD	0.27 ± 0.02	0.29 ± 0.05
E/A ratio	CON	1.37 ± 0.12	1.47 ± 0.18
	DMetD	1.39 ± 0.12	1.46 ± 0.19
e’ mean (cm/s)	CON	10.5 ± 0.2	10.4 ± 0.7
	DMetD	11.1 ± 0.5	8.9 ± 0.6
E/e’ ratio	CON	7.0 ± 0.3	7.1 ± 0.3
	DMetD	7.2 ± 0.6	8.6 ± 0.6*
DET (ms)	CON	114 ± 5	132 ± 13
	DMetD	125 ± 12	124 ± 9

CON=healthy controls (n=8), DMetD=diabetic metabolic dysfunction (n=9). LA=left atrium, Relative=normalized for body weight, LVEDD=left ventricular end diastolic diameter, PWd=posterior wall diameter, IVSd=intraventricular septum thickness end-diastole, RWT=relative wall thickness (2*PWd)/LVEDD), E=early diastolic filling velocity, E/A ratio=ratio between early and late filling velocities, e’ mean=early diastolic tissue relaxation velocity, mean of lateral and septal wall, DET=deceleration time of early diastolic filling. *p<0.05 vs CON by unpaired t-test at 5 months’ time-point. Data are mean±SEM.

Table S2. Left ventricular speckle tracking echocardiography characteristics from baseline to 5-month time points.

		BASELINE	5-MONTH
Peak rotation basal (°)	CON	0.34 ± 0.68	0.82 ± 0.53
	DMetD	1.26 ± 0.54	1.17 ± 0.41
Peak rotation apical (°)	CON	3.8 ± 0.9	5.6 ± 1.5
	DMetD	5.1 ± 2.0	3.0 ± 0.9
Peak velocity basal rotation (°/ms)	CON	5.4 ± 1.0	5.4 ± 2.0
	DMetD	5.3 ± 1.2	7.4 ± 3.4
Peak velocity apical rotation (°/ms)	CON	-27.4 ± 9.5	-35.7 ± 14.2
	DMetD	-26.7 ± 8.6	-13.3 ± 3.5
Peak twist (°)	CON	4.6 ± 1.7	4.8 ± 1.7
	DMetD	4.9 ± 2.1	2.3 ± 0.8
Time to peak twist (s)	CON	0.43 ± 0.02	0.40 ± 0.01
	DMetD	0.47 ± 0.07	0.42 ± 0.03
Peak untwist velocity (°/ms)	CON	-34 ± 9	-58 ± 17
	DMetD	-35 ± 8	-22 ± 8*†
Time to peak untwist velocity (s)	CON	0.40 ± 0.02	0.48 ± 0.02
	DMetD	0.38 ± 0.02	0.44 ± 0.02

CON=healthy controls (n=8), DMetD=diabetic metabolic dysfunction animals (n=9). *$p<0.05$ as time•diabetes interaction by two-way ANOVA, †$p<0.05$ versus CON by Bonferroni post-hoc analysis. Data are mean±SEM.

Table S3. Hemodynamic characteristics of DMetD and CON animals at sacrifice measured by Millar or Swan Ganz catheter.

	CON	DMetD	t-test
HR (bpm)	100 ± 7	85 ± 9	0.20
MAP (mmHg)	74 ± 5	88 ± 7	0.13
SAP (mmHg)	82 ± 5	95 ± 7	0.15
DAP (mmHg)	66 ± 6	79 ± 7	0.16
LV SP (mmHg)	86 ± 3	95 ± 7	0.24
LV dP/dt_{max} (mmHg s^{-1})	1323 ± 54	1286 ± 153	0.83
LV dP/dt_{min} (mmHg s^{-1})	-1181 ± 106	-1441 ± 114	0.12
LV EDP (mmHg)	9.2 ± 1.1	8.7 ± 1.3	0.76
Tau (ms)	63 ± 6	68 ± 9	0.61
DTF	0.42 ± 0.02	0.49 ± 0.03	0.11
SV (ml)	24 ± 3	27 ± 2	0.44
CO (L min^{-1})	2.4 ± 0.2	2.2 ± 0.2	0.57
SVR (mmHg L^{-1} min^{-1})	33 ± 3	42 ± 4	0.11

CON=healthy controls (n=8), DMetD=diabetic metabolic derangement animals (n=9). HR=heart rate, MAP=mean arterial pressure, SAP=systolic arterial pressure, DAP = diastolic arterial pressure, LV SP = maximal left ventricular pressure, LV dP/dt_{max}= maximum rate of rise of left ventricular pressure, LV dP/dt_{min}=maximum rate of fall of left ventricular pressure, LV EDP=left ventricular end diastolic pressure, Tau=diastolic time constant, DTF=diastolic time fraction, SV stroke volume, CO cardiac output, SVR systemic vascular resistance. Data are mean±SEM.

Table S4. Left ventricular end diastolic and end systolic volumes and pressures and stroke volume and ejection fraction based on pressure-volume loop analyses.

		Preload reduction	Baseline	Preload increase
LV EDV (ml)	CON	30 ± 5	43 ± 6	71 ± 9*
	DMetD	37 ± 4*	58 ± 6	103 ± 10*†
LV ESV (ml)	CON	7 ± 3	15 ± 3	36 ± 7*
	DMetD	16 ± 4	28 ± 5	62 ± 11*†
LV EF (%)	CON	78 ± 8	65 ± 4	49 ± 5
	DMetD	62 ± 7	54 ± 4	42 ± 7
SV (ml)	CON	23 ± 5	28 ± 4	35 ± 6
	DMetD	21 ± 2	30 ± 2	40 ± 6
LV EDP (mmHg)	CON	6.8 ± 1.7	9.7 ± 1.8	14.3 ± 2.1*
	DMetD	7.6 ± 2.0*	12.0 ± 1.9	20.0 ± 2.5*
LV ESP (mmHg)	CON	56 ± 6	68 ± 7	94 ± 9*
	DMetD	73 ± 6*	91 ± 6	113 ± 11*

CON=healthy controls (n=6), DMetD=diabetic metabolic derangement animals (n=8). All measurements were obtained during inspirational breath hold. EDV=end diastolic volume, ESV=end systolic volume, EF=ejection fraction, SV = stroke volume, EDP=end diastolic pressure, ESP end systolic pressure. *$p < 0.05$ versus corresponding baseline, †$p<0.05$ versus corresponding control by two-way ANOVA and Bonferroni post-hoc analysis. Data are mean±SEM.

Table S5. Plasma metabolic parameters of CON and DMetD animals measured at sacrifice.

	CON	DMetD	t-test
**Liver function**			
ASAT (U L^{-1})	34 ± 2	30 ± 4	0.44
ALAT (U L^{-1})	55 ± 4	17 ± 2	<0.001
**Renal function**			
Urea (mmol L^{-1})	2.9 ± 0.3	1.7 ± 0.3	0.009
Creatinine (μmol L^{-1})	111 ± 6	92 ± 8	0.07
Albumin (g L^{-1})	44 ± 1	45 ± 2	0.59

CON=healthy controls (n=8), DMetD=diabetic metabolic derangement animals (n=9); ASAT aspartate aminotransferase; ALAT alanine aminotransferase. Data are mean±SEM.

Table S6. Gene expression of significantly up- or down-regulated genes in DMetD compared to controls.

Gene-symbol	logFC	logCPM	LR	PValue	FDR
CREB3L3	4.53	1.07	16.96	3.818E-05	1.32E-02
UCP3	4.41	2.23	31.12	2.420E-08	3.24E-05
MARCO	3.93	0.12	20.40	6.274E-06	3.06E-03
ALAS2	3.54	3.76	11.78	5.973E-04	9.15E-02
CDH9	3.29	-0.65	13.52	2.359E-04	4.96E-02
AIRE	3.04	-0.13	17.97	2.239E-05	8.57E-03
MYO5B	2.98	2.52	31.74	1.767E-08	2.91E-05
HS3ST2	2.59	-0.76	12.67	3.724E-04	6.82E-02
FCN1	2.19	0.22	16.33	5.333E-05	1.68E-02
ANGPTL4	2.16	5.87	28.13	1.136E-07	1.11E-04
MYOZ1	2.16	1.35	12.71	3.637E-04	6.82E-02
PLIN2	2.15	6.59	30.91	2.709E-08	3.42E-05
BPIFC	1.96	0.12	11.96	5.428E-04	8.74E-02
SLCO6A1	1.87	0.09	12.59	3.879E-04	6.93E-02
LIPE	1.85	5.29	36.59	1.460E-09	3.91E-06
CPT1A	1.84	4.91	23.10	1.535E-06	9.97E-04
HTR4	1.71	1.65	19.03	1.288E-05	5.52E-03
RETSAT	1.56	7.95	25.37	4.739E-07	3.50E-04
PGLYRP2	1.46	0.00	12.11	5.020E-04	8.28E-02
CA4	1.44	5.40	18.13	2.063E-05	8.19E-03
RBP1	1.38	4.53	23.66	1.152E-06	7.96E-04

BTNL9	1.35	5.75	14.38	1.497E-04	3.56E-02
C7	1.25	5.95	12.97	3.163E-04	6.16E-02
LY96	1.18	2.18	11.93	5.514E-04	8.76E-02
SRPX	1.17	4.76	13.62	2.235E-04	4.74E-02
PDGFD	1.17	1.61	15.69	7.457E-05	2.16E-02
UCP2	1.04	3.37	18.89	1.388E-05	5.83E-03
CDC14A	1.01	1.00	11.62	6.512E-04	9.69E-02
ART4	1.00	3.92	16.49	4.886E-05	1.61E-02
OVOL3	-4.51	-1.98	13.44	2.461E-04	5.12E-02
CYP1A1	-2.99	3.04	12.63	3.791E-04	6.85E-02
SLC2A1	-2.25	2.99	28.70	8.437E-08	8.61E-05
STRC	-2.25	-0.35	15.83	6.917E-05	2.09E-02
GNMT	-2.19	3.45	29.78	4.850E-08	5.20E-05
SLC16A6	-2.15	0.99	20.59	5.675E-06	2.83E-03
ALDH4A1	-1.87	6.19	41.10	1.445E-10	1.03E-06
SLC26A6	-1.79	1.65	17.53	2.832E-05	1.03E-02
MCCC2	-1.78	4.35	27.56	1.525E-07	1.42E-04
AASS	-1.72	3.90	20.35	6.456E-06	3.08E-03
CYP4F55	-1.65	5.32	37.87	7.547E-10	2.70E-06
IZUMO4	-1.61	3.37	26.66	2.428E-07	2.08E-04
SQLE	-1.54	1.73	12.21	4.752E-04	7.96E-02
HAPLN3	-1.50	5.23	48.09	4.062E-12	8.71E-08
CYP4F2	-1.50	3.01	45.52	1.514E-11	1.62E-07
BDH1	-1.49	3.90	19.43	1.044E-05	4.66E-03

ADHFE1	-1.45	7.07	36.23	1.755E-09	4.18E-06
SYCE2	-1.42	4.12	18.45	1.740E-05	7.17E-03
BCKDHB	-1.40	5.60	29.83	4.722E-08	5.20E-05
WBSCR27	-1.38	0.75	17.01	3.711E-05	1.31E-02
MUT	-1.34	5.30	31.26	2.263E-08	3.23E-05
CBX8	-1.33	0.75	12.37	4.365E-04	7.49E-02
MPND	-1.33	5.11	20.64	5.532E-06	2.83E-03
CAMKV	-1.32	1.21	13.37	2.563E-04	5.28E-02
MCCC1	-1.31	5.64	31.38	2.124E-08	3.23E-05
MOCS1	-1.31	3.57	20.63	5.579E-06	2.83E-03
GCDH	-1.29	3.03	25.70	3.989E-07	3.17E-04
ALDH6A1	-1.27	6.98	36.99	1.189E-09	3.64E-06
CCT6B	-1.25	0.98	12.22	4.737E-04	7.96E-02
RANGRF	-1.24	5.50	25.07	5.516E-07	3.94E-04
SLC25A29	-1.24	5.65	34.75	3.752E-09	7.31E-06
PC	-1.19	3.17	12.30	4.519E-04	7.69E-02
GNB3	-1.02	4.04	14.86	1.160E-04	2.96E-02

The genes differentially affected by diabetes. LogFC=log fold change, logCPM=log counts per million, LR=likelihood ratio, FDR=false discovery rate. Absolute logFC > 1 with a P-value < 0.001, indicating a minimal 2 fold change in gene expression; FDR<0.1.

Figure S1. **(**related to Figure 3) Expression of the N2BA and N2B in the myocardium of CON and DMetD swine. The samples have been applied in triplicate to the gel and the results averaged. Outlined are the sections as shown in Figure 3C in the main article.

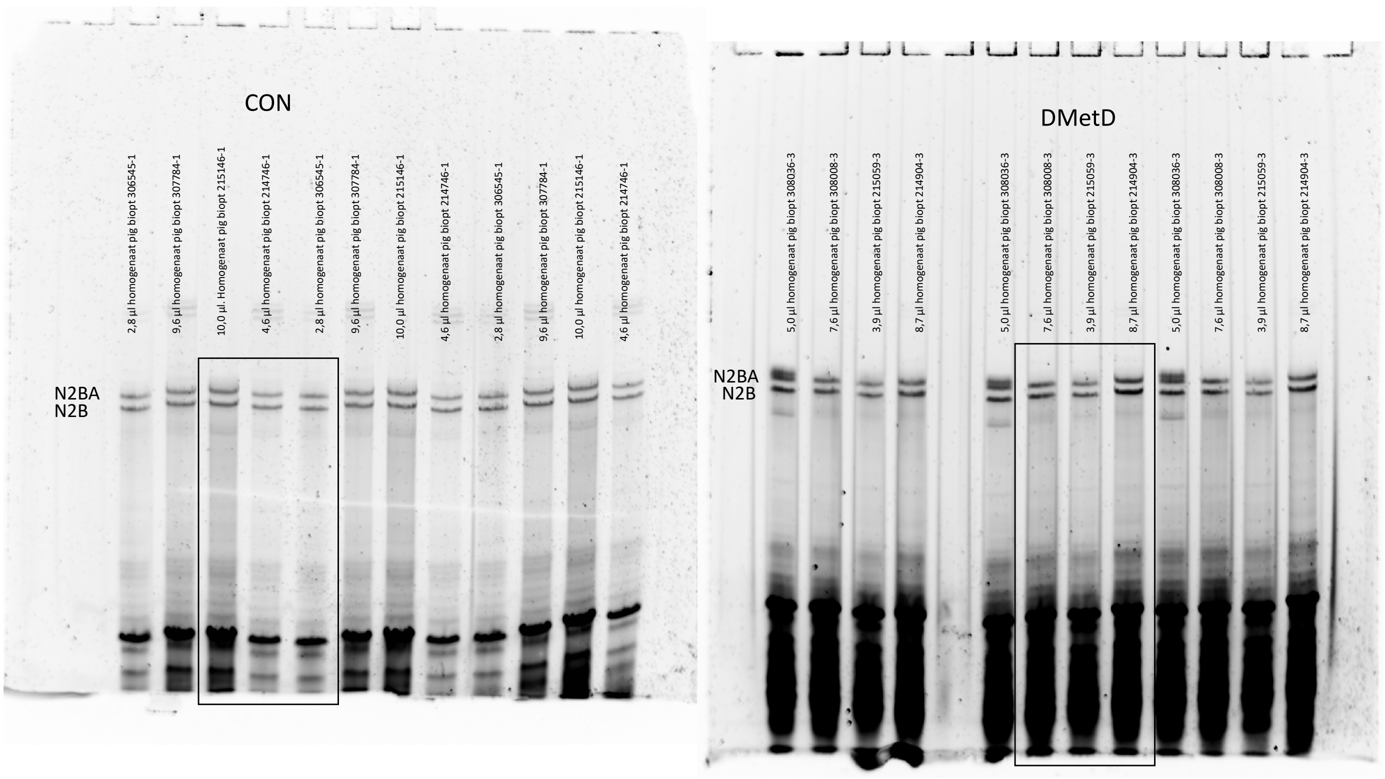

Figure S2. **(**related to Figure 4) Full length gel blots of eNOS proteins. Outlined are the sections as shown in Figure 4C-E in the main article.

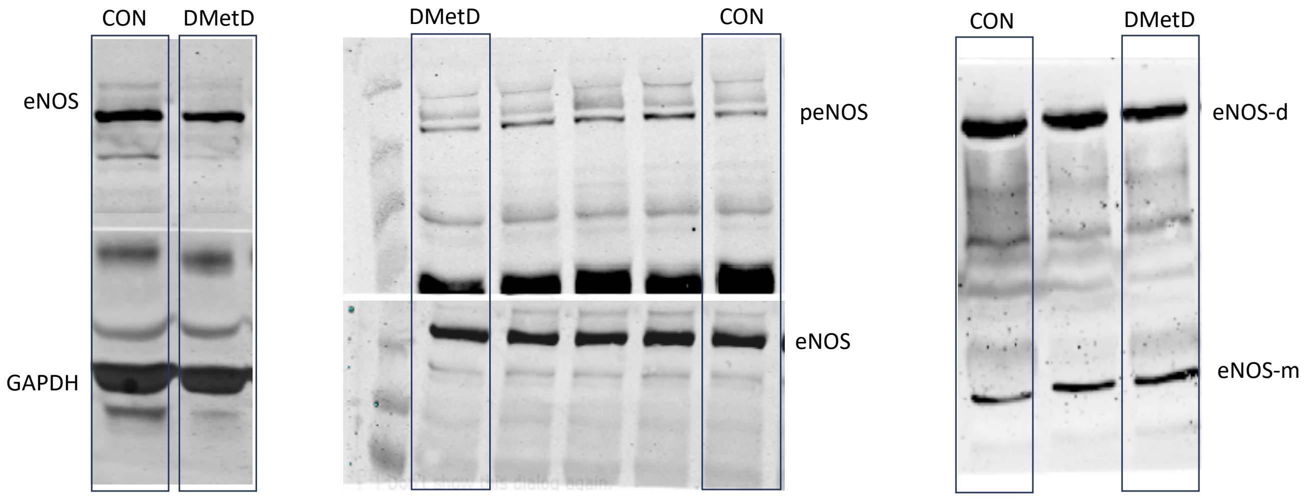

Figure S3 (related to Figure 5G). Mitochondrial protein concentrations in the myocardium of CON and DMetD swine.

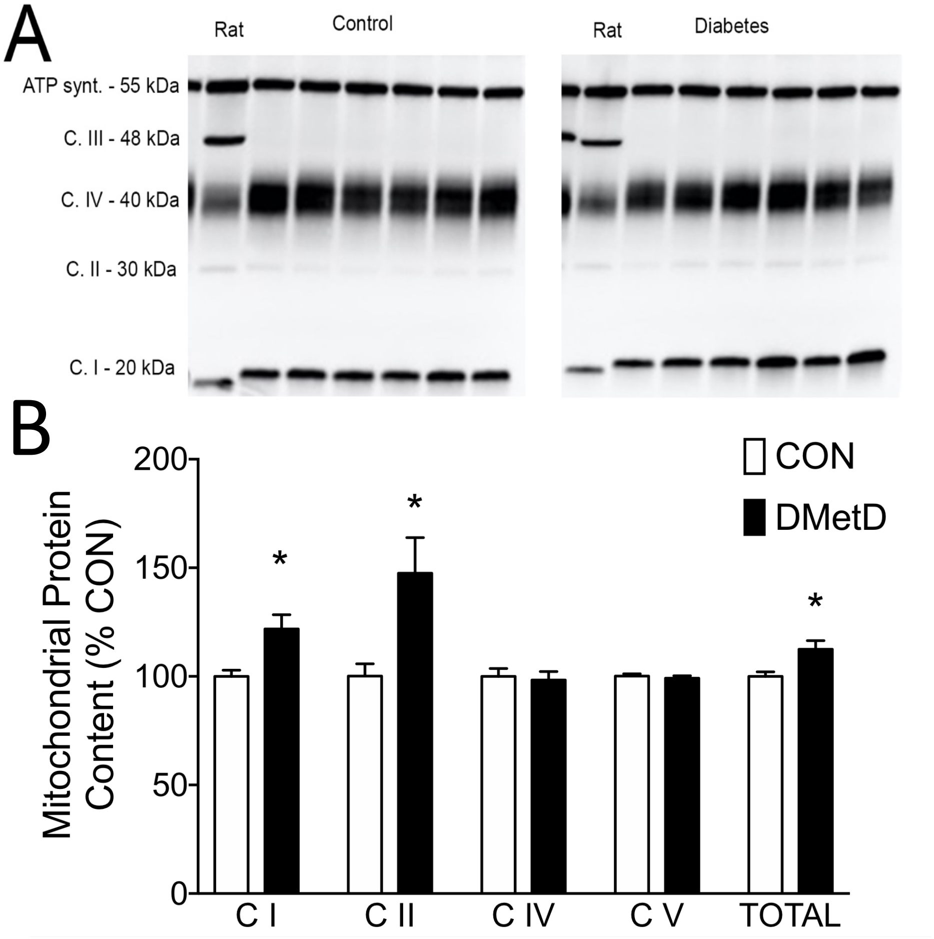

A. Typical example of a Western blot with an antibody cocktail against five complexes: complex I (C.I) subunit NDUFB8 at 20 kDa, complex II (C.II) subunit at 30kDa, complex III (C. III) core protein 2 at 48 kDa, complex IV (C.IV) subunit I at 40 kDa and ATP synthase (ATP synt) α-subunit. Note that C.III did not cross-react between rats and pigs. Samples from different blots were normalized based on a reoccurring rat sample and protein concentration. **B:** Total mitochondrial protein content was higher in DMetD, which was due to higher protein concentration of mitochondrial complex I and II subunits only. n=6 each group, in duplo. *: $P<0.05$ *vs.* CON.

Figure S4. (related to Figure 5H), Cardiac mitochondrial respiration is lower in DMetD compared to CON.

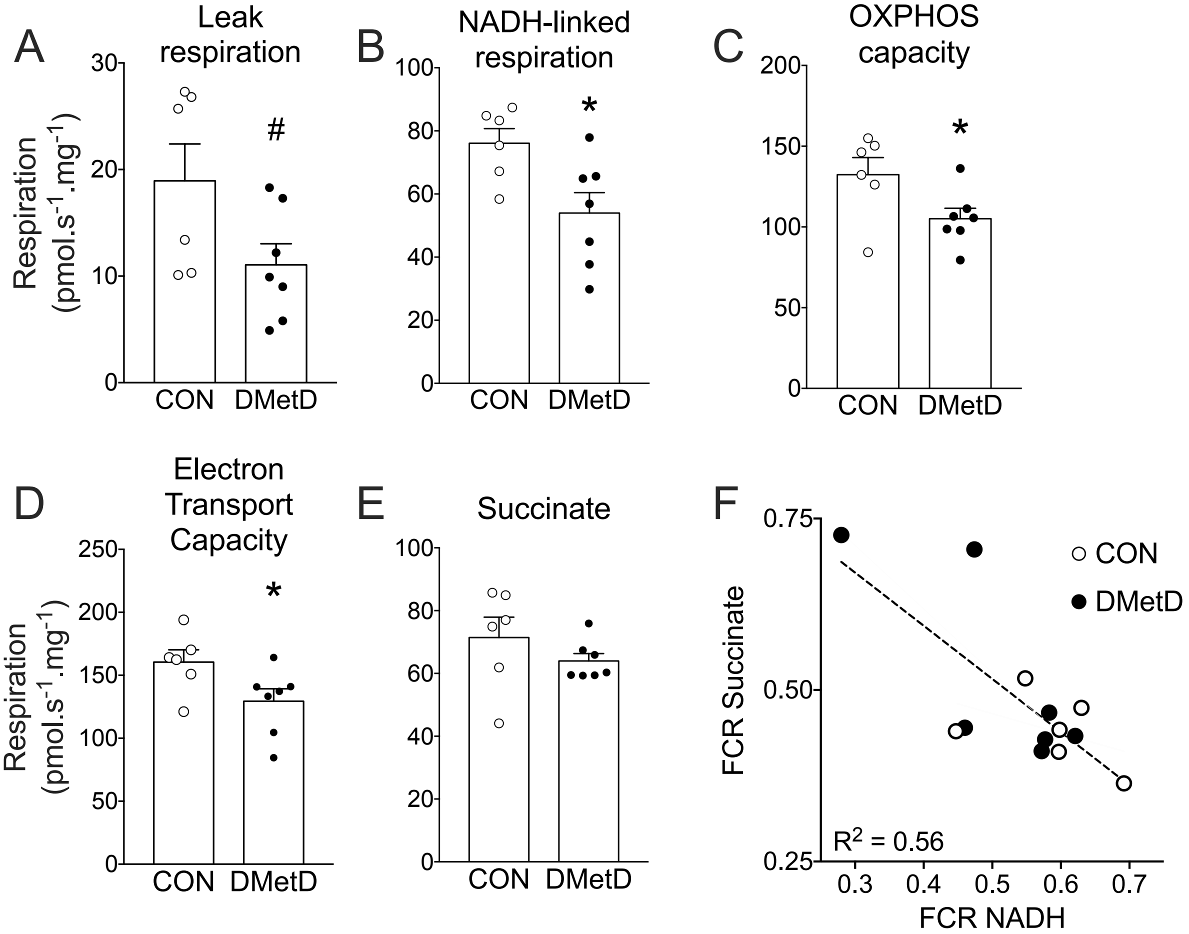

High-resolution respirometry was performed in permeabilized cardiac fibers from 6 CON and 7 DMetD hearts. **A**: Leak respiration with NADH-substrates (glutamate, pyruvate and malate) tended to be lower in DMetD compared to CON (#: P=0.06). NADH-linked respiration (**B**), OXPHOS capacity (with additional succinate, **C**) and electron transport capacity (**D**) were significantly lower in DMetD. Succinate-linked respiration via complex II, under the presence of the complex I blocker rotenone (**E**) was not different between groups (P=0.22), indicative that the lower maximal respiration was due to NADH-linked, complex I, dysfunction. The normalized flux (flux control ratio; FCR) for NADH-linked respiration correlated with the FCR for succinate, indicative that complex II partly compensates for the lower NADH-linked respiration in DMetD. Measurements performed in duplo and averaged. *: P<0.05 *vs.* CON

Figure S5. **(**related to Figure 6) Full length gel blots of AMPK proteins. Outlined are the sections as shown in Figure 6B, C in the main article.

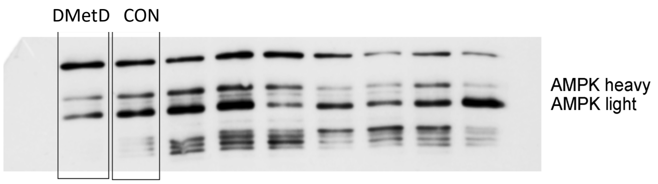

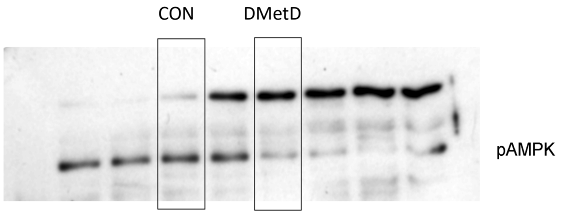

Supplement: Supplementary file 1 — Supplementary Information. [file 41598_2020_68637_MOESM1_ESM.pdf]
